# The association between retinal microvasculature derived from optical coherence tomography angiography and systemic factors in type 2 diabetics

**DOI:** 10.3389/fmed.2023.1107064

**Published:** 2023-03-13

**Authors:** Yi Li, Kunfang Wu, Zilin Chen, Guihua Xu, Dingding Wang, Juanjuan Wang, Gabriella Bulloch, Grace Borchert, Huiya Fan

**Affiliations:** ^1^Department of Ophthalmology, Huizhou Central People’s Hospital, Huizhou, China; ^2^Shantou University Medical College, Shantou, China; ^3^Centre for Eye Research Australia, Melbourne, VIC, Australia; ^4^The University of Melbourne, Melbourne, VIC, Australia

**Keywords:** OCTA, retinal microvasculature, systemic factors, T2DM, correlation

## Abstract

**Aims:**

To investigate the correlation between the retinal microvasculature using optical coherence tomography angiography (OCTA) and systemic factors in type 2 diabetes mellitus (T2DM) patients.

**Methods:**

This cross-sectional study obtained OCTA data from patients with T2DM administered at hospital and referred to ophthalmic services. Patient data about demographics, comorbid conditions, and blood biomarkers were extracted from electronic medical records. Data from OCTA scans obtained by CIRRUS HD-OCT Model 5,000 were obtained. Vessel density (VD) and perfusion density (PD) within the superficial capillary plexus, and foveal avascular zone (FAZ) area were automatically segmented. These parameters were tested for their correlations with systemic factors by univariate and multivariable linear regression analyses.

**Results:**

A total of 144 T2DM patients (236 eyes) were available for analysis, with mean age of 53.6 (SD = 10.34) and 56.9% were male. Chronic kidney disease, cardiovascular disease, increased serum creatinine (Scr), red blood cell count (RBC), platelets (PLT), apolipoprotein B (APOB), and decreased urine albumin to creatinine ratio (UACR) were significantly associated with lower VD and PD (all *p* < 0.013). UACR and triglyceride (TRIG) were significantly correlated with FAZ area (all *p* < 0.017). In multivariate analyses, PLT, eGFR, and APOB were independent risk factors for retinal rarefaction, and UACR was a significant predictor of FAZ area.

**Conclusion:**

We found several systemic risk factors, such as PLT, renal function and lipid profiles were associated with PD, VD, and FAZ area among Chinese T2DM patients.

## Introduction

Type 2 diabetes mellitus (T2DM) is a worldwide epidemic that carries considerable morbidity, mortality, and financial burden from its deleterious complications and associations with other comorbid conditions. According to the latest International Diabetes Federation (IDF) diabetes atlas (1), an estimated 537 million people had diabetes in 2021, with this figure projected to reach 643 million by 2030.

Type 2 diabetes mellitus accounts for over 90% of all diabetes worldwide (1, 2) and is characterized by chronic hyperglycemia and insulin resistance resulting from lifestyle and genetic factors. If uncontrolled, T2DM leads to vascular damage of the eyes, kidneys, and heart. (3) Increased vascular permeability, vascular cell apoptosis, and altered blood flow contribute to macrovascular (peripheral vascular disease and coronary heart disease) and microvascular (diabetic retinopathy and diabetic nephropathy) complications (4) which result in morbidity and eventually mortality if unmanaged. Therefore, early identification and risk stratification of T2DM patients who are at risk of vascular complications is an area of growing research for the control and prevention of poor outcomes.

The retina is a structure at the back of the eye that contains a rich network of microvasculature. Growing evidence suggests retinal imaging can detect microstructural changes to vascular networks, (5) and fundoscopy studies (6–8) report concordance between the retinal microvasculature and systemic risk factors such as hypertension, diabetes, and smoking. A recent study also discovered significant retinal microvascular alterations in diabetic patients with subclinical atherosclerosis. (9) These findings have led to the idea that the retina is the window to the cardiovascular system and its suggestion as a screening tool.

Optical coherence tomography angiography (OCTA) is a non-invasive imaging technique that allows for three-dimensional visualization of retinal microvasculature networks with contrast for high-resolution imaging. Unlike fundoscopy, it can detect subtle microvascular abnormalities on retinal layers and choriocapillaris, which has led to its establishment for the early detection of diabetic retinopathy (DR). (10, 11) Additionally, OCTA can quantify the number of perfused vessels in the vascular bed (functional rarefaction) and perfused vessels in the tissue (structural rarefaction) (12), making it a useful tool for evaluating microvascular changes longitudinally in people with T2DM, dyslipidemia, and chronic kidney disease. (13–15)

Despite the widespread use of OCTA for eye diseases, little is known about the impact of systemic risk factors on OCTA parameters in diabetic eyes. Therefore, this study investigated the association between OCTA-derived retinal microvasculature parameters and systemic factors to understand its impact on vascular function in a Chinese diabetic population.

## Materials and methods

### Study population

This cross-sectional study included T2DM patients who had admitted to and received ophthalmic consultation in Huizhou Central People’s Hospital from January 2021 to June 2022. This study was approved by the Institutional Review Board of Huizhou Central People’s Hospital (IRB approval number: kyl20210115) and followed the tenets of the Declaration of Helsinki. Written informed consent was obtained from all participants.

This study included patients with T2DM (2) aged >18 years old. Participants were excluded if they had: (1) severe media opacity (e.g., corneal disease, dense cataract, vitreous hemorrhage); (2) any ocular illness that may affect ocular circulation (e.g., glaucoma, retinal vascular occlusion, retinal detachment, exudative aged macular degeneration, pathologic myopia); (3) signal strength of OCTA scans <5/10, or OCTA scans with artifacts or segmentation errors; (4) a history of surgical treatments for eye diseases (except cataract) or laser treatment; (5) uncontrollable high blood pressure (HBP) (≥180/110 mmHg); (6) any severe systemic diseases (e.g., tumor, heart failure, and cerebral infarction);

### Obtaining data on systemic factors and blood biomarkers

Systemic factors were retrieved from patient electronic medical records (EMR) and included gender, age, time from diagnosis of T2DM, body mass index (BMI), blood pressure readings, smoking history, cardiovascular disease history, chronic kidney disease history, obesity, and blood biomarkers. These included systolic blood pressure (SBP), diastolic blood pressure (DBP), glucose, hemoglobin A1c (HbA1c), red blood cell count (RBC), hemoglobin (HGB), blood platelet (PLT), serum creatinine (Scr), estimated glomerular filtration rate (eGFR), urine albumin to creatinine ratio (UACR), total cholesterol (CHOL), triglyceride (TRIG), high-density lipoprotein cholesterol (HDL), low-density lipoprotein cholesterol (LDL), lipoprotein a (Lpa), apolipoprotein A (ApoA), apolipoprotein B (ApoB). All patients had their blood drawn at 8 AM after an overnight fast and before taking morning medications. Overnight first-void urine samples were also obtained. The eGFR was calculated based on the Chronic Kidney Disease Epidemiology Collaboration (CKD-EPI) equation (16). Chronic kidney disease was defined as eGFR<60 ml/min/1.73m^2^. Body mass index was calculated as weight in kilograms divided by the square of height in meters. Obesity was defined as BMI ≥ 28 kg/m^2^.

### Ocular examinations and imaging

**Figure 1:**
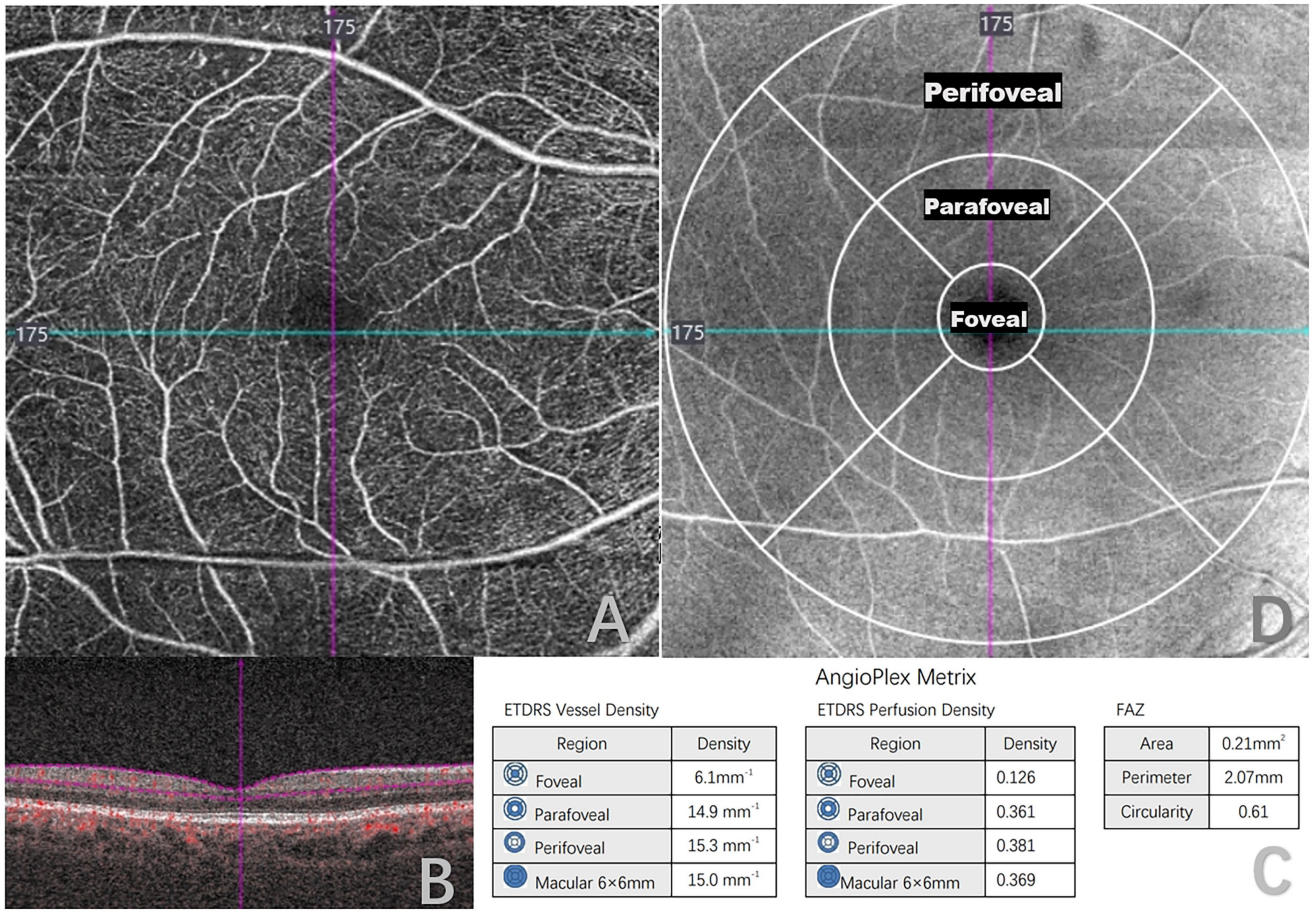
Quantitative measurement of optical coherence tomography angiography (OCTA) 6 × 6-mm scans in a type 2 diabetes mellitus (T2DM) patient. **(A)** 6 × 6-mm en face image of the superficial capillary plexus (SCP). **(B)** B-scans with flow encoding show the slab segmentation (horizontal purple lines), which included the SCP. **(C)** Angioplex metrics, including vessel density, perfusion density and foveal avascular zone (FAZ) parameters. **(D)** OCT en face image of the superficial layer overlaid with the early treatment of diabetic retinopathy study grid (ETDRS).

All patients underwent an ophthalmic examination, which included best-corrected visual acuity, intraocular pressure, silt lamp examination, fundus photographs, fluorescein fundus angiography (FFA), optical coherence tomography (OCT), and OCTA by a single trained technician. The presence of DR was confirmed based on FFA, and was categorized as NDR, mild non-proliferative DR (mild NPDR), moderate non-proliferative DR (moderate NPDR), severe non-proliferative DR (severe NPDR), and proliferative DR (PDR) according to the International Clinical Diabetic Retinopathy Severity Scales. (17) Patients underwent OCTA scanning using CIRRUS HD-OCT Model 5,000 (Carl Zeiss, Germany), which uses a super luminescent diode (SLD) with a central wavelength of 840 nm, and a scanning speed of 68,000 A-scans/s. The macular region was scanned using a 6 mm × 6 mm scan pattern, each consisting of 245 A-scan per B-scan. This was automatically divided into three fields: the foveal area (a central circle with a diameter of 1 mm), the parafoveal area (an annulus centered on the fovea with an inner ring with a diameter of 3 mm), and the perifoveal area (an annulus centered on the fovea with outer ring diameters of 6 mm; Figure 1).Vessel density (VD), perfusion density (PD), and foveal avascular zone (FAZ) parameters were quantitatively analyzed within the superficial capillary plexus (SCP), defined as the area extending from the inner limiting membrane to 110 μm above the retinal pigment epithelium. This was analyzed by built-in angiography software, which calculated the average VD and PD using a grid overlay according to standard ETDRS subfields. VD was defined as the total length of perfused vessels per unit area in the measurement region, and PD was defined as the total area of perfused retinal microvasculature per unit area on binarized vasculature images. FAZ was defined as a region within the foveal at the center of the retina devoid of retinal blood vessels. Area, perimeter, and circularity are FAZ parameters that we used for this study.

### Statistical analysis

All data analyses were performed using SPSS version 25.0 (IBM Corp, Armonk, NY, USA). Continuous data were represented as mean ± standard deviations (SD), categorical data were expressed as number (percentage, %). Univariate linear regression models were used to analyze potential associations between systemic risk factors and OCTA-derived metrics, with regression coefficients calculated to estimate the magnitude of microvascular change associated with predictor variables. Bonferroni correction for multiple comparison was performed to assess differences between FAZ parameters and VD, PD at each annulus. Multiple linear regression analyses were subsequently performed to determine independent risk factors of retinal microvascular dysfunction. Generalized estimating equations approach were used to adjust for correlations between paired eyes. A *p*-value of <0.013 (0.05/4) for VD, PD, and a *p*-value of <0.017 (0.05/3) for FAZ parameters were considered statistically significant for association.

## Results

A total of 191 patients underwent OCTA examinations. Thirty participants were excluded due to a history of reported ocular diseases, surgeries, or laser treatments, and 10 participants were excluded due to a history of severe systemic diseases or having type 1 diabetes. A further seven participants were excluded due to poor quality. 236 eyes of 144 T2DM patients were included for analysis, with a mean (SD) age of 53.61 (10.34) years, and 56.9% males. Characteristics of participants are detailed in Table 1, and prevalence of hypertension (33.3%), chronic kidney disease (12.5%), smoking history (22.2%), obesity (7.6%), and cardiovascular disease (6.3%) were noted amongst study subjects.

**Table 1:** Patient demographics and clinical characteristics.

Characteristic	Subjects (*n* = 144)
**Demographics**	
Male, *n* (%)	82 (56.9)
Age (y)	53.61 ± 10.34
DM duration (y)	7.92 ± 5.48
BMI (kg/m^2^)	23.51 ± 3.39
SBP (mmHg)	128.36 ± 16.51
DBP (mmHg)	80.19 ± 10.04
**Comorbidities**	
Hypertension, *n* (%)	48 (33.3)
Chronic kidney disease, *n* (%)	18 (12.5)
Cardiovascular disease, *n* (%)	9 (6.3)
Smoking history, *n* (%)	32 (22.2)
Obesity, *n* (%)	11 (7.6)
DR, *n* (%)	115 (79.9)
**DR stage**	
Mild NPDR, *n* (%)	54 (20.1)
Moderate NPDR, *n* (%)	16 (11.1)
Severe NPDR, *n* (%)	22 (15.3)
PDR, *n* (%)	23 (16.0)
**Lab values**	
HbA1c (%)	9.69 ± 2.56
Glucose (mmol/L)	12.13 ± 5.69
HGB (g/L)	133.40 ± 19.30
RBC (10^12/L)	4.59 ± 0.77
PLT (10^9/L)	254.01 ± 73.72
Scr (μmol/L)	72 (IQR 61–88)
eGFR (mL/min/L.73m^2^)	89.05 ± 24.84
UACR (mg/g)	19 (IQR 8–89)
CHOL (mmol/L)	4.84 ± 1.23
TRIG (mmol/L)	2.34 ± 1.98
HDL (mmol/L)	1.00 ± 0.30
LDL (mmol/L)	3.05 ± 1.07
Lpa (mg/L)	0.29 ± 0.27
APOA (g/L)	1.19 ± 0.26
APOB (g/L)	1.12 ± 0.57

BMI, body mass index; SBP, systolic blood pressure; DBP, diastolic blood pressure; DM, diabetes mellitus; DR, diabetic retinopathy; NPDR, nonproliferative diabetic retinopathy; PDR, proliferative diabetic retinopathy; RBC, red blood cell count; HGB, hemoglobin; PLT, blood platelet; eGFR, estimated glomerular filtration rate; Scr, serum creatinine; UACR, urine albumin to creatinine ratio; CHOL, total cholesterol; TRIG, triglyceride; HDL, high-density lipoprotein cholesterol; LDL, low-density lipoprotein cholesterol; Lpa, lipoprotein a; ApoA, apolipoprotein A; ApoB, apolipoprotein B.

Table 2 describes the mean characteristics of vessel density and perfusion density within regions captured by OCTA. Mean SCP-VD in the parafoveal region was 14.97 ± 2.88 and 15.23 ± 2.31 mm^−1^ in the macular region. Mean SCP-PD in the parafoveal region was 0.36 ± 0.07 and 0.37 ± 0.06 in the macular region. The average FAZ area was 0.29 ± 0.12 mm^2^.

**Table 2:** Vessel density and perfusion density in superficial vascular capillary plexus and foveal avascular zone (FAZ) measurements.

Optical coherence tomography angiography (OCTA) parameters	(*n* = 236 eyes)
Signal strength	7.78 ± 1.28
**Vessel density (mm^−1^)**	
Foveal	5.76 ± 2.91
Parafoveal	14.97 ± 2.88
Perifoveal	15.67 ± 2.26
Macular 6^*^6 mm	15.23 ± 2.31
**Perfusion density**	
Foveal	0.13 ± 0.07
Parafoveal	0.36 ± 0.07
Perifoveal	0.39 ± 0.06
Macular 6^*^6 mm	0.37 ± 0.06
**FAZ parameters**	
FAZ area (mm^2^)	0.29 ± 0.12
FAZ perimeter (mm)	2.25 ± 0.55
FAZ circularity	0.68 ± 0.09

**Figure 2:**
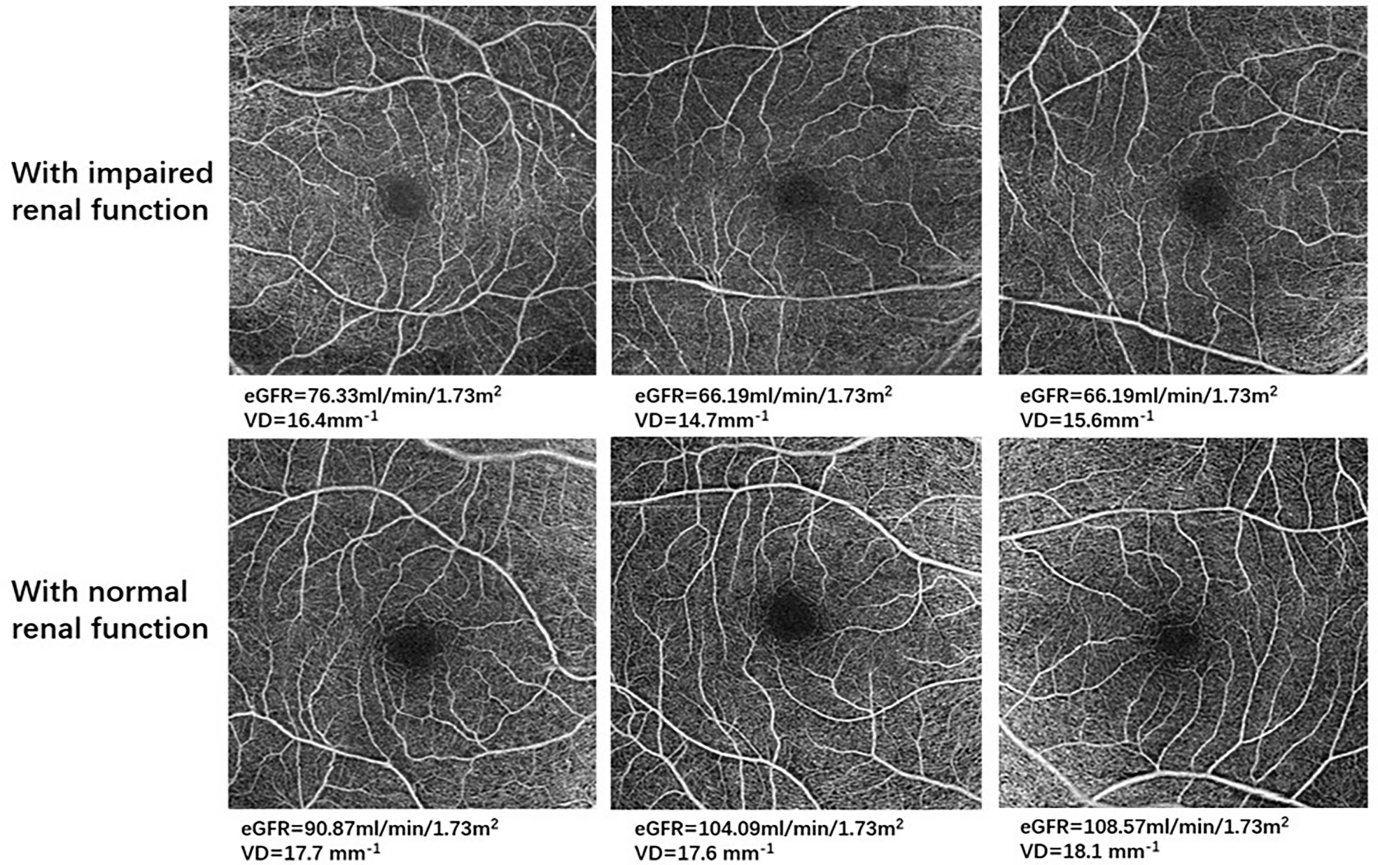
Optical coherence tomography angiography (OCTA) images in representative patients with impaired renal function and with normal renal function. For all 6 × 6-mm OCTA images, the top row demonstrates retinal microvasculature in type 2 diabetes mellitus (T2DM) patients with impaired renal function, and the bottom row shows retinal microvasculature in T2DM patients with normal renal function.

Table 3 demonstrates the association of various systemic factors with SCP-VD in anatomical regions captured by OCTA. Significant associations with signal strength, sex, cardiovascular disease, DR stage, CKD, RBC, PLT, Scr, UACR, and APOB for VD were apparent on univariate analysis (all *p* < 0.05). Following multivariable analysis, macular VD correlated positively with signal strength (*β* = 0.968, *p* < 0.001), eGFR (*β* = 0.601, *p* = 0.009), and APOB (*β* = 0.290, *p* < 0.001).Similarly, abnormal renal function was associated with reduced VD as measured by OCTA (Figure 2). Foveal VD was also significantly correlated with signal strength (*β* = 0.559, *p* = 0.001), diabetes mellitus (DM) duration (*β* = 0.576, *p* = 0.011), and PLT (*β* = 0.544, *p* = 0.003).

**Table 3:** Associations of systemic factors with vessel density in 6 × 6-mm optical coherence tomography angiography (OCTA) scans.

	(a) Univariate analysis							
	Foveal	*P*-value	Parafoveal	*P*-value	Perifoveal	*P*-value	Macular	*P*-value
**Demographics**								
Sex	**1.157**	**0.007** ^*^	0.394	0.370	0.091	0.798	0.191	0.596
Age	−0.298	0.117	−0.088	0.790	−0.197	0.302	−0.176	0.370
DM duration	0.212	0.420	−0.187	0.298	**−0.337**	**0.045**	−0.289	0.072
BMI	−0.007	0.977	0.391	0.079	0.121	0.543	0.177	0.365
SBP	0.238	0.463	0.026	0.904	−0.294	0.177	−0.211	0.309
DBP	0.379	0.148	0.279	0.209	−0.014	0.947	0.061	0.763
Signal strength	0.285	0.136	**0.931**	**<0.001** ^*^	**0.981**	**<0.001** ^*^	**0.949**	**<0.001** ^*^
**Comorbidities**								
Hypertension	−0.507	0.293	−0.459	0.358	−0.526	0.174	−0.512	0.200
Cardiovascular disease	0.050	0.950	0.837	0.108	**1.061**	**0.011** ^*^	**0.988**	**0.019**
Smoking	0.542	0.384	**0.906**	**0.044**	**0.800**	**0.048**	**0.817**	**0.037**
Chronic kidney disease	0.921	0.452	**−1.234**	**0.046**	**−1.626**	**0.009** ^*^	**−1.469**	**0.013**
Obesity	−0.025	0.969	0.764	0.311	0.316	0.660	0.411	0.563
**DR stage**								
Mild NPDR	0.192	0.702	−0.754	0.115	−0.756	0.037	−0.724	0.053
Moderate NPDR	−0.202	0.708	−0.812	0.162	−0.652	0.138	−0.685	0.132
Severe NPDR	1.304	0.171	**−1.744**	**0.031**	**−2.220**	**<0.001** ^*^	**−2.018**	**0.002** ^*^
PDR	0.555	0.438	**−1.982**	**0.001** ^*^	**−2.229**	**<0.001** ^*^	**−2.107**	**<0.001** ^*^
**Lab values**								
Glucose	0.003	0.986	−0.044	0.814	−0.019	0.897	−0.025	0.867
HbA1c	0.077	0.744	0.134	0.524	0.063	0.714	0.082	0.642
HGB	−0.055	0.868	**0.586**	**0.018**	**0.437**	**0.031**	**0.459**	**0.025**
RBC	0.171	0.406	**0.472**	**0.011** ^*^	**0.347**	**0.039**	**0.372**	**0.025**
PLT	**0.565**	**0.008** ^*^	0.342	0.059	0.263	0.079	0.288	0.057
Scr	**0.872**	**0.011** ^*^	−0.017	0.923	−0.221	0.413	−0.145	0.538
eGFR	−0.447	0.187	0.184	0.363	**0.417**	**0.036**	0.343	0.072
UACR	0.641	0.188	−0.468	0.148	**−0.590**	**0.011** ^*^	**−0.532**	**0.030**
CHOL	0.411	0.080	0.005	0.981	−0.023	0.889	−0.004	0.981
TRIG	0.006	0.971	−0.188	0.360	−0.211	0.184	−0.200	0.222
HDL	−0.245	0.236	−0.148	0.461	0.095	0.532	0.031	0.842
LDL	0.340	0.231	−0.010	0.957	−0.041	0.819	−0.024	0.892
Lpa	0.256	0.564	−0.037	0.811	−0.071	0.713	−0.057	0.732
APOA	−0.180	0.415	−0.170	0.422	−0.060	0.722	−0.088	0.608
APOB	**0.411**	**0.012** ^*^	**0.427**	**<0.001** ^*^	**0.312**	**0.001** ^*^	**0.341**	**<0.001** ^*^
**(b) Multivariable analysis**
	**Foveal**	***P*-value**	**Parafoveal**	***P*-value**	**Perifoveal**	***P*-value**	**Macular**	***P*-value**
Signal strength	**0.559**	**0.001** ^*^	**1.008**	**<0.001** ^*^	**0.973**	**<0.001** ^*^	**0.968**	**<0.001** ^*^
DM duration	**0.576**	**0.011** ^*^	0.208	0.316	0.082	0.329	0.123	0.404
Hypertension	**−0.911**	**0.033**	−0.023	0.955	−0.043	0.875	−0.059	0.837
**DR stage**										(b) Multivariable analysis		Foveal	*P*-value	Parafoveal	*P*-value	Perifoveal	*P*-value	Macular	*P*-value
Mild NPDR	−0.009	0.993	−0.986	0.152	**−1.283**	**0.017**	**−1.193**	**0.028**
Moderate NPDR	0.727	0.401	−0.628	0.408	−1.045	0.066	−0.904	0.123
Severe NPDR	−0.355	0.487	−0.831	0.118	−0.634	0.090	−0.681	0.077
PDR	0.033	0.947	−0.792	0.084	**−0.768**	**0.015**	**−0.750**	**0.023**
PLT	**0.544**	**0.003** ^*^	0.182	0.277	0.095	0.413	0.124	0.313
eGFR	−0.077	0.817	0.547	0.064	**0.637**	**0.005** ^*^	**0.601**	**0.009** ^*^
CHOL	0.235	0.355	0.433	0.061	**−0.324**	**0.038**	**−0.327**	**0.040**
APOB	0.099	0.449	**0.424**	**0.001** ^*^	**0.258**	**0.001** ^*^	**0.290**	**<0.001** ^*^

Values in bold are results that are statistically significant before Bonferroni correction (*P* < 0.05).

^*^Statistically significant results (*P* < 0.013).

Multivariable model-adjusted for age, sex, signal strength, DM duration, hypertension, cardiovascular disease, DR stage, chronic kidney disease, RBC, PLT, eGFR, UACR, CHOL, TRIG, and APOB.

**Figure 3:**
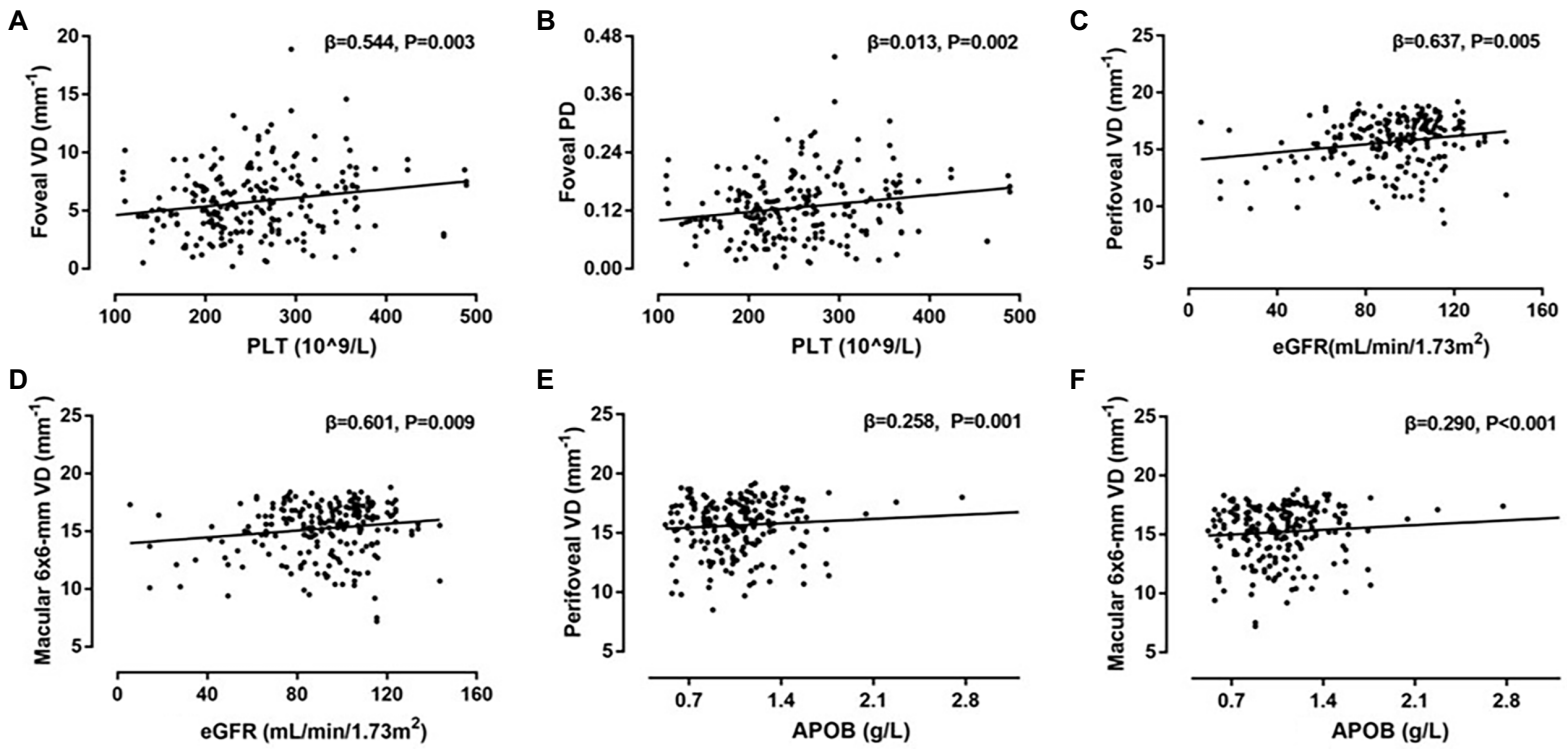
Correlations between systemic risk factors and optical coherence tomography angiography (OCTA) parameters. **(A)** Scatter plot between platelets (PLT) and vessel density (VD) in foveal; **(B)** scatter plot between PLT and perfusion density (PD) in foveal; **(C)** scatter plot between eGFR and VD in perifoveal; **(D)** scatter plot between eGFR and VD in macular 6 × 6-mm; **(E)** scatter plot between APOB and VD in perifoveal; **(F)** Scatter plot between apolipoprotein B (APOB) and VD in macular 6 × 6-mm.

Table 4 demonstrates associations between systemic factors and SCP-PD. Univariate linear regression analysis showed that sex, signal strength, cardiovascular disease, DR stage, PLT, Scr, and APOB were associated with PD (all *p* < 0.013). Following adjustment for confounding factors, positive associations remained for signal strength (*β* = 0.027, *p* < 0.001), DM duration (*β* = 0.013, *p* = 0.012), PLT (*β* = 0.013, *p* = 0.002), eGFR (*β* = 0.017, *p* = 0.006), and APOB (*β* = 0.007, *p* = 0.002). The diagrams showing the correlations between OCTA parameters and systemic risk factors such as PLT, APOB and eGFR are shown in Figure 3.

**Table 4:** Associations of systemic factors with perfusion density in 6 × 6-mm optical coherence tomography angiography (OCTA) scans.

	(a) Univariate analysis							
	Foveal	*P*-value	Parafoveal	*P*-value	Perifoveal	*P*-value	Macular	*P*-value
**Demographics**								
Sex	**0.027**	**0.007** ^*^	0.013	0.248	0.006	0.504	0.008	0.371
Age	−0.007	0.133	−0.002	0.700	−0.005	0.281	−0.005	0.350
DM duration	0.005	0.391	−0.004	0.422	**−0.009**	**0.041**	−0.007	0.075
BMI	−0.001	0.843	0.009	0.108	0.003	0.596	0.004	0.419
SBP	0.006	0.453	0.001	0.910	−0.008	0.165	−0.006	0.300
DBP	0.009	0.149	0.007	0.238	−0.001	0.887	0.001	0.823
Signal strength	0.007	0.139	**0.023**	**<0.001** ^*^	**0.026**	**<0.001** ^*^	**0.025**	**<0.001** ^*^
**Comorbidities**								
Hypertension	−0.011	0.301	−0.011	0.382	−0.013	0.203	−0.013	0.229
Cardiovascular disease	0.001	0.945	0.020	0.152	**0.027**	**0.011** ^*^	**0.024**	**0.025**
Smoking	0.013	0.378	0.022	0.056	**0.021**	**0.049**	**0.021**	**0.040**
Chronic kidney disease	0.027	0.374	−0.025	0.117	**−0.037**	**0.027**	**−0.033**	**0.036**
Obesity	−0.004	0.805	0.014	0.461	0.003	0.859	0.006	0.750
DR stage								
Mild NPDR	0.005	0.674	−0.018	0.142	−0.019	0.050	−0.018	0.064
Moderate NPDR	−0.003	0.827	−0.016	0.272	−0.015	0.209	−0.015	0.211
Severe NPDR	0.032	0.157	−0.035	0.089	**−0.051**	**0.003** ^*^	**−0.045**	**0.010** ^*^
PDR	0.019	0.272	**−0.040**	**0.012** ^*^	**−0.047**	**0.001** ^*^	**−0.004**	**0.001** ^*^
**Lab values**								
Glucose	0.000	0.989	−0.001	0.765	−0.001	0.865	−0.001	0.835
HbA1c	0.002	0.768	0.002	0.680	0.002	0.675	0.002	0.669
HGB	−0.002	0.797	**0.014**	**0.033**	**0.011**	**0.033**	**0.012**	**0.032**
RBC	0.003	0.542	**0.011**	**0.024**	**0.009**	**0.043**	**0.009**	**0.035**
PLT	**0.013**	**0.008** ^*^	0.008	0.067	0.007	0.089	0.007	0.068
Scr	**0.021**	**0.019**	0.001	0.821	−0.005	0.440	−0.003	0.579
eGFR	−0.011	0.170	0.003	0.501	0.010	0.051	0.008	0.100
UACR	0.017	0.160	−0.010	0.243	**−0.014**	**0.030**	−0.012	0.064
CHOL	0.010	0.078	−0.001	0.910	−0.001	0.823	−0.001	0.895
TRIG	0.000	0.969	−0.005	0.323	−0.005	0.224	−0.005	0.248
HDL	−0.005	0.250	−0.004	0.378	0.001	0.705	0.000	0.995
LDL	0.008	0.216	0.000	0.963	−0.001	0.802	−0.001	0.870
Lpa	0.007	0.507	0.000	0.985	−0.001	0.825	−0.001	0.864
APOA	−0.004	0.408	−0.005	0.364	−0.002	0.602	−0.003	0.517
APOB	**0.009**	**0.019**	**0.010**	**0.001** ^*^	**0.007**	**0.008** ^*^	**0.008**	**0.004** ^*^
**(b) Multivariable analysis**
	**Foveal**	***P*-value**	**Parafoveal**	***P*-value**	**Perifoveal**	***P*-value**	**Macular**	***P*-value**
Signal strength	**0.014**	**<0.001** ^*^	**0.026**	**<0.001** ^*^	**0.027**	**<0.001** ^*^	**0.027**	**<0.001** ^*^
DM duration	**0.013**	**0.012** ^*^	0.005	0.300	0.001	0.801	0.002	0.563
Hypertension	**−0.021**	**0.029**	−0.001	0.911	−0.002	0.782	−0.002	0.748
**DR stage**								
Mild NPDR	0.005	0.831	−0.018	0.327	−0.021	0.142	−0.020	0.174		(b) Multivariable analysis		Foveal	*P*-value	Parafoveal	*P*-value	Perifoveal	*P*-value	Macular	*P*-value
Moderate NPDR	0.021	0.201	−0.010	0.606	−0.020	0.192	−0.017	0.283
Severe NPDR	−0.006	0.617	−0.017	0.203	−0.014	0.155	−0.015	0.146
PDR	0.001	0.905	−0.020	0.088	**−0.020**	**0.016**	**−0.019**	**0.023**
PLT	**0.013**	**0.002** ^*^	0.004	0.295	0.002	0.557	0.003	0.405
eGFR	−0.001	0.857	0.014	0.052	**0.018**	**0.003** ^*^	**0.017**	**0.006** ^*^
CHOL	0.006	0.312	−0.012	0.043	**−0.009**	**0.023**	**−0.009**	**0.023**
APOB	0.002	0.535	**0.010**	**0.002** ^*^	**0.006**	**0.004** ^*^	**0.007**	**0.002** ^*^

Values in bold are results that are statistically significant before Bonferroni correction (*P* < 0.05).

^*^Statistically significant results (*P* < 0.013).

Multivariable model-adjusted for age, sex, signal strength, DM duration, hypertension, cardiovascular disease, DR stage, chronic kidney disease, RBC, PLT, eGFR, UACR, CHOL, TRIG, and APOB.

Table 5 shows the association between systemic factors and FAZ parameters in OCTA 6 × 6-mm scans. Univariate linear regression analysis showed that DR stage, UACR, and TRIG were associated with FAZ parameters (all *p* < 0.017). In a multivariable-adjusted model, UACR was negatively associated with FAZ area (*β* = −0.029, *p* = 0.007) and FAZ perimeter (*β* = −0.159, *p* = 0.003), whilst age and chronic kidney disease positively impacted FAZ area (*β* = 0.030, *p* = 0.06; *β* = 0.128, *p* = 0.016, respectively), and FAZ perimeter (*β* = 0.117, *p* = 0.014; *β* = 0.688, *p* = 0.007, respectively).

**Table 5:** Association of systemic factors with FAZ parameters in OCTA 6 × 6-mm scans.

	Univariate						Multivariable-adjusted					
	FAZ area	*P*-value	FAZ perimeter	*P*-value	FAZ circularity	*P*-value	FAZ area	*P*-value	FAZ perimeter	*P*-value	FAZ circularity	*P*-value
**Demographics**												
Sex	0.022	0.245	0.053	0.525	0.016	0.186	0.017	0.358	0.037	0.666	0.008	0.520
Age	**0.017**	**0.044**	0.061	0.125	0.006	0.276	**0.030**	**0.006** ^*^	**0.117**	**0.014** ^*^	−0.003	0.707
DM duration	−0.001	0.932	0.000	0.996	−0.005	0.360	-	-	-	-	-	-
BMI	0.002	0.483	−0.006	0.612	0.001	0.738	-	-	-	-	-	-
SBP	−0.009	0.342	−0.041	0.336	−0.006	0.340	-	-	-	-	-	-
DBP	−0.012	0.180	−0.058	0.107	−0.008	0.230	-	-	-	-	-	-
Signal strength	0.003	0.722	0.009	0.804	0.006	0.232	-	-	-	-	-	-
**Comorbidities**												
Hypertension	−0.005	0.803	0.001	0.989	−0.003	0.809	0.018	0.399	0.083	0.358	0.004	0.758
Cardiovascular disease	−0.011	0.711	−0.015	0.900	0.019	0.140	-	-	-	-	-	-
Smoking	0.000	0.999	−0.002	0.982	−0.002	0.884	-	-	-	-	-	-
Chronic kidney disease	−0.007	0.854	0.054	0.767	**−0.045**	**0.017**	**0.128**	**0.016** ^*^	**0.688**	**0.007** ^*^	−0.072	0.018
Obesity	0.031	0.167	−0.130	0.272	0.029	0.234	-	-	-	-	-	-
DR stage												
Mild NPDR	**−0.052**	**0.010** ^*^	**−0.211**	**0.011** ^*^	−0.017	0.215	**−0.063**	**0.009** ^*^	**−0.259**	**0.010** ^*^	−0.016	0.282
Moderate NPDR	0.021	0.448	**0.210**	**0.046**	**−0.065**	**0.004** ^*^	0.028	0.343	**0.249**	**0.034**	**−0.070**	**0.005** ^*^
Severe NPDR	−0.021	0.481	−0.155	0.277	−0.021	0.190	0.017	0.679	−0.067	0.731	−0.002	0.906
PDR	−0.065	0.067	−0.247	0.128	**−0.052**	**0.018**	−0.025	0.514	−0.067	0.674	−0.038	0.187
**Lab values**												
Glucose	−0.013	0.118	−0.046	0.159	−0.003	0.648	-	-	-	-	-	-
HbA1c	−0.013	0.153	−0.034	0.394	−0.007	0.165	-	-	-	-	-	-
HGB	−0.005	0.670	−0.013	0.773	−0.003	0.617	-	-	-	-	-	-
RBC	−0.005	0.607	−0.010	0.800	0.001	0.822	-	-	-	-	-	-
PLT	−0.016	0.123	−0.063	0.150	−0.004	0.503	-	-	-	-	-	-
Scr	−0.015	0.104	−0.057	0.148	−0.002	0.646	-	-	-	-	-	-
eGFR	0.009	0.371	0.032	0.460	0.001	0.916	0.027	0.059	0.113	0.069	−0.012	0.270
UACR	**−0.024**	**<0.001** ^*^	**−0.125**	**0.001** ^*^	−0.001	0.897	**−0.029**	**0.007** ^*^	**−0.159**	**0.003** ^*^	0.005	0.375
CHOL	**−0.020**	**0.020**	**−0.083**	**0.026**	0.002	0.699	−0.014	0.074	−0.051	0.128	0.002	0.718
TRIG	**−0.017**	**0.007** ^*^	−0.057	0.102	−0.008	0.248	−0.008	0.239	−0.026	0.438	−0.010	0.170
HDL	0.006	0.528	0.007	0.862	0.011	0.119	-	-	-	-	-	-
LDL	−0.013	0.152	−0.057	0.156	−0.001	0.854	-	-	-	-	-	-
Lpa	0.007	0.447	0.029	0.585	−0.004	0.668	-	-	-	-	-	-
APOA	0.004	0.720	−0.008	0.862	0.007	0.351	-	-	-	-	-	-
APOB	−0.007	0.216	−0.033	0.124	**0.008**	**0.026**	-	-	-	-	-	-

Values in bold are results that are statistically significant before Bonferroni correction (*P* < 0.05).

^*^Statistically significant results (*P* < 0.017).

Multivariable model-adjusted for age, sex, DM duration, hypertension, DR stage, chronic kidney disease, eGFR, UACR, CHOL, and TRIG.

## Discussion

In this study, the retinal microvasculature of a Chinese population with T2DM was examined for its correlation with systemic factors. Of note, several blood biomarkers and systemic influences were correlated with VD and PD regions of interest within OCTA scans after adjustment for confounding factors, including signal strength, DM duration, PLT, eGFR, and APOB. After multivariable analysis, age, chronic kidney disease, DR stage, UACR, and APOB correlated with FAZ parameters. Our results suggest the retinal microvasculature may be influenced by the presence of systemic factors.

### DM duration correlates with OCTA parameters

DM duration was independently associated with foveal VD and PD in the multivariable-adjusted model, indicating the long-term impact of abnormal blood glucose levels in the microvascular system. Our findings were in concordance with previous research by Czakó et al. (18), who found that DM duration was strongly associated with decreased retinal VD after interaction analysis with the effects of systemic risk factors, and by Qian et al. (11), who reported a negative correlation between DM duration and OCTA metrics such as SCP-VD and SCP-PD in 1118 DM patients. Furthermore, larger FAZ and lower retinal capillary densities in children and adolescents with diabetes were observed in a case–control study (19), and these changes are associated with DM duration and poor glycemic control.

Although DM duration was a significant risk factor for microvascular abnormalities, we found no correlations between OCTA parameters and HbA1c or blood glucose in univariate or multivariable models. In this study, we assessed T2DM patients with a relatively short period of diabetes (71.5%, ≤10 years), and less than half of the patients (43.3%) had poor glycemic control (HbA1c > 10%), which may not be representative of all disease durations, and the results should be interpreted with caution.

### Hypertension weakly correlates with OCTA parameters

Hypertension negatively impacted foveal VD and PD after controlling for confounding factors (*p* < 0.05), demonstrating some influence over vessel integrity. However, none of these correlations persist after Bonferroni correction. In spite of several observational studies (20, 21) not finding hypertension or blood pressure to be risk factors for microvascular complication in diabetics, multiple OCTA studies have demonstrated its impacts on retinal microvasculature, including Lee et al. (13) whom reported hypertension correlated with lower SCP-VD (*β* = −0.239, *p* = 0.039) in diabetic patients than hypertensive controls, and case-control studies by Sun et al. (22) and Donati et al. (23) demonstrating non-diabetic hypertensive eyes had decreased VD as well as increased FAZ after adjusting for sex, age, and ocular parameters. In addition, a longitudinal analysis of 4,758 T2DM patients with non- or mild DR demonstrated blood pressures conferred to risk of DR progression (24).

Hypertension is thought to contribute to accelerated microvascular impairment in individuals with T2DM. Chronic hyperglycemia results in global microvascular changes like thickening of the vascular basement membrane and increased endothelial permeability, and the presence of hypertension increases pressure along these membranes which accelerate the pathological change and weaken retinal capillary walls. Therefore, a deficit in perfusion density on OCTA should present as a red flag for underlying poor blood pressure control and could be a risk factor if investigated further. More studies with large-scale sample sizes and detailed blood pressure monitoring are required to clarify the impact of hypertension on retinal microvasculature and diabetes management.

### Chronic kidney disease and renal function correlate with OCTA parameters

Our results showed that eGFR was positively associated with VD and PD, which was in line with results from previous studies exploring correlations between renal function and retinal microvasculature. Yeung et al. (25) reported that patients with CKD (eGFR<60 mL/min/1.73m^2^) had lower parafoveal SCP-VD compared to those of control group (*p* < 0.001), with eGFR strongly related with SCP-VD in multivariate-adjusted models. Observational cross-sectional studies (26, 27) aimed at investigating the relationship between systemic risk factors and OCTA parameters in patients with systemic hypertension found a significant correlation between eGFR and retinal capillary density after adjusting for age, sex, and blood pressure, suggesting impaired renal function could be one of important risk factors in retinal microvascular alterations. Similarly, Zhuang et al. (28) demonstrated that decreased SCP-VD was independently correlated with lower eGFR among T2DM patients, while other investigators (29) found a significant relationship between lower SCP-VD, SCP-PD, and higher UACR in T2DM patients after controlling for systemic and ocular parameters.

In addition, our study showed that chronic kidney disease positively impacted FAZ area and perimeter, while UACR was negatively associated with FAZ area and perimeter after adjusting for multiple variables. Lee et al. (13) reported that lower eGFR was associated with greater FAZ size in diabetic patients, which suggested that abnormal renal function may have an impact on the foveal and adjacent small vessels. However, FAZ morphology can be variable even in healthy individuals (30, 31), this variation must be considered and posed as a challenge when assessing possible pathological FAZ alternations. A relatively low number of chronic kidney disease patients (18/144) in our study population may hinder the interpretation of these findings, larger longitudinal studies will be needed to examine the effects of renal function in OCTA-derived metrics.

### Aberrant lipid indices correlate with OCTA parameters

Our study suggested that APOB was positively correlated with parafoveal, perifoveal and macular VD and PD, after controlling for other variables. TRIG was negatively correlated with FAZ area, although this correlation did not persist in multivariable analysis.

Dyslipidemia is an established risk factor for microvascular complications. It is now recognized that elevated CHOL levels induced inflammatory reaction in the microvascular system, which occurs long before events in the large vessels. (32) A randomized placebo-controlled trial by Kaushik et al. (33) proved that cholesterol-reducing medications retards DR progression in diabetic patients with proper glycemic control and hypercholesterolemia. This observation corresponds well with a nested case–control study by Aryan et al. (34) whom indicated a positive association of serum CHOL levels with microvascular complications (OR = 1.1, CI:1.0–2.2, *p* = 0.004) on 444 T2DM cases and 439 controls, although this correlation disappeared after interaction analysis with demographic and systemic factors. A large-scale cohort study (35), on the other hand, found a significant correlation between elevated serum levels of TRIG, decreased HDL levels, and diabetes-related microvascular complications in 72,289 T2DM patients, implying that aberrant lipid indices may reflect retinal microangiopathy in diabetics.

While there is little evidence that LDL has a causal effect on the risk of microvascular disease, growing evidence (36, 37) has shown that compared to traditional lipid indices, ApoB provides incremental information on lipid metabolism and may play a significant role in the development of vascular disease. To date, only a few studies have looked into the relationship between ApoB and retinal vascular system in diabetics. Shi et al. (38) found that foveal SCP-VD measured from OCTA 3 × 3 mm scans were negatively correlated with serum ApoB levels in T2DM patients (*β* = −0.016, *p* < 0.001), however, this correlation was not significant after controlling for other risk factors.

### PLT correlates with OCTA parameters

Our study found that PLT was significantly associated with increased VD and PD in the foveal region after adjusting for other confounders. The influence of PLT on the microvascular system has so far remained uncertain. Considering the physical proximity of PLT to the vascular endothelium, a relationship between PLT and microvascular alterations is assumed. Yuan et al. (39) implicated that platelet hyperactivity in diabetic individuals may undermine tissue perfusion as well as contribute to microvascular occlusion. Data from 3,009 participants recruited for the Blue Mountains Eye Study (BMES) (40) revealed that higher PLT correlated with narrower arteriolar caliber and wider venular caliber, implying that elevated levels of PLT could have adverse effects on microvasculature. However, the mechanisms that underlie this association are unclear and research on this topic is sparse. Based on OCTA measurement, we speculate that PLT levels may be a marker for microvascular dysfunction in diabetic patients. More studies are required to corroborate this hypothesis.

### Limitation

There are several limitations of our present study. The first one is that the study was a single-center study with a relatively small sample size. Second, most participants in this study have mild or moderate diabetic retinopathy (115/140, 79.9%), while the effect of diabetic retinopathy has been taken into account in multivariable models, it may still have confounding effects on OCTA measurement due to the pathological change in DR itself. Thirdly, we did not account for ocular factors, such as axial length and refractive error in the analysis, as subjects with high myopia (axial length > 26 mm) were excluded. However, ocular magnification in OCTA images caused by varying axial lengths may interfere with accurate interpretation of OCTA measures. (10) Finally, VD and PD in the deep capillary plexus (DCP) could not be evaluated due to the limitations of built-in angiography software in the OCTA instrument, which may be more sensitive in detecting retinal microvascular changes in diabetic patients at an early stage.

In conclusion, this study provided evidence that systemic risk factors are associated with retinal microvasculature among T2DM patients in a Chinese population. Further longitudinal and large-scale studies are needed to corroborate our findings.

## Data availability statement

The original contributions presented in the study are included in the article/supplementary material, further inquiries can be directed to the corresponding author.

## Ethics statement

The studies involving human participants were reviewed and approved by the Institutional Review Board of Huizhou Central People’s Hospital. The patients/participants provided their written informed consent to participate in this study.

## Author contributions

YL and KW drafted the manuscript and interpreted the results. YL, GX, and KW performed the data analysis. HF, ZC, GX, DW, and JW revised the manuscript for important intellectual content. GBu and GBo were involved in interpreting the results. All authors contributed to the article and approved the submitted version.

## Funding

This study was supported by the Science and Technology Plan Project of Huizhou Science and Technology Bureau (grant number: 2021WC0106369).

## Conflict of interest

The authors declare that the research was conducted in the absence of any commercial or financial relationships that could be construed as a potential conflict of interest.

## Publisher’s note

All claims expressed in this article are solely those of the authors and do not necessarily represent those of their affiliated organizations, or those of the publisher, the editors and the reviewers. Any product that may be evaluated in this article, or claim that may be made by its manufacturer, is not guaranteed or endorsed by the publisher.
